# Using Regional Homogeneity to Reveal Altered Spontaneous Activity in Patients with Mild Cognitive Impairment

**DOI:** 10.1155/2015/807093

**Published:** 2015-02-09

**Authors:** Yumei Wang, Xiaochuan Zhao, Shunjiang Xu, Lulu Yu, Lan Wang, Mei Song, Linlin Yang, Xueyi Wang

**Affiliations:** ^1^Department of Psychiatry, The First Hospital of Hebei Medical University, Shijiazhuang 050031, China; ^2^Institute of Mental Health, Hebei Medical University, Shijiazhuang 050031, China

## Abstract

Most patients with mild cognitive impairment (MCI) are thought to be in an early stage of Alzheimer's disease (AD). Resting-state functional magnetic resonance imaging reflects spontaneous brain activity and/or the endogenous/background neurophysiological process of the human brain. Regional homogeneity (ReHo) rapidly maps regional brain activity across the whole brain. In the present study, we used the ReHo index to explore whole brain spontaneous activity pattern in MCI. Our results showed that MCI subjects displayed an increased ReHo index in the paracentral lobe, precuneus, and postcentral and a decreased ReHo index in the medial temporal gyrus and hippocampus. Impairments in the medial temporal gyrus and hippocampus may serve as important markers distinguishing MCI from healthy aging. Moreover, the increased ReHo index observed in the postcentral and paracentral lobes might indicate compensation for the cognitive function losses in individuals with MCI.

## 1. Introduction

Mild cognitive impairment (MCI) is an intermediate stage in the cognitive changes of normal aging and the fully developed clinical features of dementia [1]. Approximately 50% of patients with MCI, particularly amnestic MCI, will develop Alzheimer's disease (AD) within 3–5 years [1, 2]. Thus, MCI has an important association with AD, and it is often considered a prodromal phase of AD. The earlier identification of MCI has been the goal of numerous aging and dementia studies, and this research has attracted much attention.

Resting-state functional magnetic resonance imaging (fMRI) signals identify spontaneous neuronal activity [3, 4] and/or the endogenous/background neurophysiological process of the human brain [5–7]. Convergent evidence has suggested that resting-state fMRI is a primary method of mechanism detection, diagnostic assessment, or therapeutic monitoring of MCI and AD [7–9]. Thus, there has been increasing interest in the use of resting-state fMRI in AD- and MCI-related studies [10–22].

Regional homogeneity (ReHo), proposed by Zang et al. [23], can evaluate the similarity between the time series of a given voxel and its nearest neighbors, rapidly map the level of regional activity across the whole brain [24], and provide an effective measurement of brain activity. It has been widely used to investigate the impaired spontaneous brain activity in several brain disorders, such as attention deficit hyperactivity disorder [25], schizophrenia [26], Parkinson's disease [27], and depression [28]. For AD and MCI, there are also several studies that have investigated the altered regional spontaneous activity patterns in resting state [16, 17, 29–31]. For the first study, He and colleagues found that AD patients showed significant impaired ReHo indices in the posterior cingulated cortex/precuneus (PCC/PCu), the bilateral cuneus, left lingual gyrus, and right fusiform gyrus, and they also found that the ReHo value in PCC/Pcu was correlated with the disease progression measured by the minimental state examination (MMSE) scores [17]. Bai and colleagues proposed that impairment in the PCC/PCu could be an important marker for distinguishing amnestic-type mild cognitive impairment (aMCI) from healthy aging [29]. Zhang and colleagues found altered spontaneous activities in the left inferior parietal lobule (IPL), medial prefrontal cortex, and PCC/PCu in individuals with MCI [30]; more importantly for the first time, they found that the ReHo indices are the good features to classify patients from normal healthy subjects. Furthermore, Liu and colleagues found that AD patients had disease severity-related reduction of ReHo indices in several regions of the default mode network [31]. The above studies all detected altered spontaneous activities in subjects with MCI or AD, strengthening the need for another independent ReHo study of MCI.

The aims of the present study were to investigate the spontaneous activity patterns of a group of early MCI subjects and to replicate the study using the ReHo index. Thus, 30 subjects with MCI and 32 subjects with normal cognitive function were included in the study. First, the ReHo index was calculated for each subject, and then two-sample two-tailed* t*-tests were performed after controlling forage and gender effects. The correlation between the ReHo indices of the identified regions and the cognitive variables (measured by the minimental state examination [MMSE] and Montreal cognitive assessment [MoCA]) were then computed to investigate the relationship between the spontaneous activity and clinical variables in the subjects with MCI.

## 2. Materials and Methods

### 2.1. Subjects

All patients and volunteers were recruited by posting advertisements in outpatient service and evaluated by trained mental health workers at the First Hospital of Hebei Medical University. Participants were selected by the database of a previous study [32]. Our study was authorized by the Ethics Committee of the First Hospital of Hebei Medical University and all participants gave written informed consent. The participants did not take any medication that might influence cognition during the MRI data scans. Each subject was right-handed and underwent a battery of neuropsychological tests, including the minimental state examination (MMSE), Montreal cognitive assessment (MoCA) and activities of daily living scale (ADL). The diagnostic and included/excluded criteria for all the subjects were based on our previous study [32].

In brief, the diagnostic criteria for MCI were identical to the criteria used by Petersen et al. [1] and included the following factors: (1) memory complaint lasting ≥6 months; (2) an activities of daily living scale score is less than 26; (3) normal general cognitive function, as indicated by an MMSE score between 20 and 27 (illiterate, ≤20; primary school, ≤23; and secondary school and above, ≤27); Montreal cognitive assessment score less than 26; (4) no dementia, as determined by the DSM-IV (The Diagnostic and Statistical Manual of Mental Disorders) criteria.

The following served as the criteria for NC: (1) no memory complaints, (2) an ADL score larger than 26, (3) normal cognitive function, and (4) no dementia, as determined by the DSM-IV criteria.

The following exclusion criteria were applied: (1) concomitant use of psychotropic medication in large quantity or loss cognitive ability to cooperate with the study or metabolic conditions, such as hypothyroidism or vitamin B12 or folic acid deficiencies; (2) combined with other brain disorders, such as schizophrenia, Parkinsonian syndrome, depression, epilepsy, or other brain diseases that influence cognitive function; (3) with a metallic foreign body, such as cochlear implants, heart stents, or other relevant contraindications for MRI; and (4) infarction or brain hemorrhage. (5) The demographic and neuropsychological details for the included subjects are shown in Table 1.

### 2.2. Data Acquisition

All the MR images were acquired with a 3.0 T Philips MR system at the second Hospital of Hebei Medical University using a standard head coil. The resting-state fMRI scans were performed using an echo planar imaging (EPI) sequence with scan parameters of repetition time = 2000 ms, echo time = 30 ms, flip angle = 90°, matrix = 64 × 64, field of view = 220 mm × 220 mm, and each volume comprised 33 slices with slice thickness = 5 mm without slice gap. The scantime last for 6 minute 40 seconds with 200 time points were obtained for each subject. During the fMRI scans, all subjects were instructed to keep their eyes closed and to relax and move as little as possible. Tight foam padding was used so as to reduce the subjects' head motion, and ear-plugs were used to reduce noise. For each subject, T1- and T2-weighted images were collected during the scan. All of the images were evaluated by two senior radiologists to exclude the subjects with microvascular or severe atrophy.

### 2.3. Data Preprocessing

All preprocessing steps were performed using the Brainnetome fMRI toolkit (brat, http://www.brainnetome.org/brat), which was developed based on statistical parametric mapping (SPM8, http://www.fil.ion.ucl.ac.uk/spm). Detailed processing steps have been reported [30]. Briefly, (1) slice timing (reference slice is 17) was performed to correct the acquisition time delay between the different slices after the first 10 volumes of each functional time series were discarded from the analysis to allow for magnetization equilibrium and to adapt the subjects to the scanning situation; (2) realignment to the first volume, according to the estimated head movement, was performed to correct for geometrical displacements; (3) spatially normalized to the standard EPI template and resampled to 2 mm × 2 mm × 2 mm; (4) denoise with several sources of spurious variances including the estimated motion parameters, linear drift, average time series in the cerebrospinal fluid and white matter regions were regressed out using general linear model; (5) temporal filtering with bandpass filter (0.01–0.08 Hz) was performed to reduce the effect of low-frequency drifts and high-frequency uninteresting noise signals; and (6) the filtered images were smoothed with a 6 mm full width at half maximum Gaussian kernal to reduce the spatial noise.

Head motion parameters were computed by estimating the translation in each direction and the angular rotation on each axis for each volume. Anyone who had a maximum displacement in any of the cardinal directions (*x*, *y*, *z*) > 3 mm or a maximum spin (*x*, *y*, *z*) > 3° was excluded. No subject was excluded for none of them has large head motion. Detailed statistical head motion parameters are showed in Table 1.

### 2.4. ReHo Measure

A detailed definition of ReHo was reported by Zang et al. [23]. In short, the ReHo index is calculated using Kendall's coefficient of concordance (KCC) [33]. For a given voxel, $\text{ReHo}=\left(\sum {\left(R_{i}\right)}^{2}-n{\left(\overset{-}{R}\right)}^{2}\right)/\left(K^{2}\left(n^{3}-n\right)/12\right)$, where *R*_*i*_ = ∑_*j*=1_^*k*^ *r*_*ij*_ is the sum rank of the* i*th time point, and *r*_*ij*_ is the rank of the *i*th time point of the* j*th voxel; $\overset{-}{R}$ is the mean of the *R*_*i*_,* n* is the length of the time series, and *k* is the number of voxels (*k* = 27 in the present study) within the measured cluster. The ReHo index ranges from 0 to 1; a higher ReHo index correlates to greater similarity between the local activity of a given voxel and that of its neighbors. We normalized the ReHo value for each subject to reduce the effect of individual variability [27, 30, 34]; that is means for each voxel, ReHo = ReHo(*x*, *y*, *z*)/mean(ReHo).

### 2.5. Statistical Analysis

A two-sample two-tailed* t*-test was performed to identify the differences between the MCI and NC groups with age and gender as covariates. The resultant *T* value map was then thresholded using *P* < 0.01 (*T* = 2.392,* df* = [1,58]) for each voxel and a cluster size of at least 50 voxels (uncorrected).

Subsequently, the identified brain regions demonstrating significant differences were extracted as regions of interest (ROI), and the mean ReHo values were used for a* posthoc* analysis after controlling for age and gender effects. Statistical comparisons of the mean adjusted ReHo values were performed using a two-sample two-tailed* t*-test at a threshold of *P* < 0.05 (FDR corrected, with the number of significant brain regions).

To evaluate if the ReHo index changes are disease severity-related in the MCI patients, correlation analyses were performed for the mean ReHo index and the clinical cognitive ability variables (e.g., MMSE and MoCA) in the MCI group at *P* < 0.05 (uncorrected statistical significance level).

## 3. Results

The demographic and psychological characteristics of the 30 patients with MCI (12 males, 18 females; mean age, 69.1 ± 5.8 years) are summarized in Table 1. The 32 healthy volunteer individuals (17 males, 15 females; mean age, 70.1 ± 5.5 years) were well matched with the MCI group in age (*P* = 0.49, two-sample two-tailed* t*-test) and gender (*P* = 0.30, Chi-squared test). The clinical variables (e.g., MMSE and MoCA) were significantly lower in the MCI subjects compared with the volunteers with normal cognitive function (Table 1). The head motion parameters did not demonstrate significant changes in the MCI subjects compared with the NC volunteers (Table 1).

**Figure 1:**
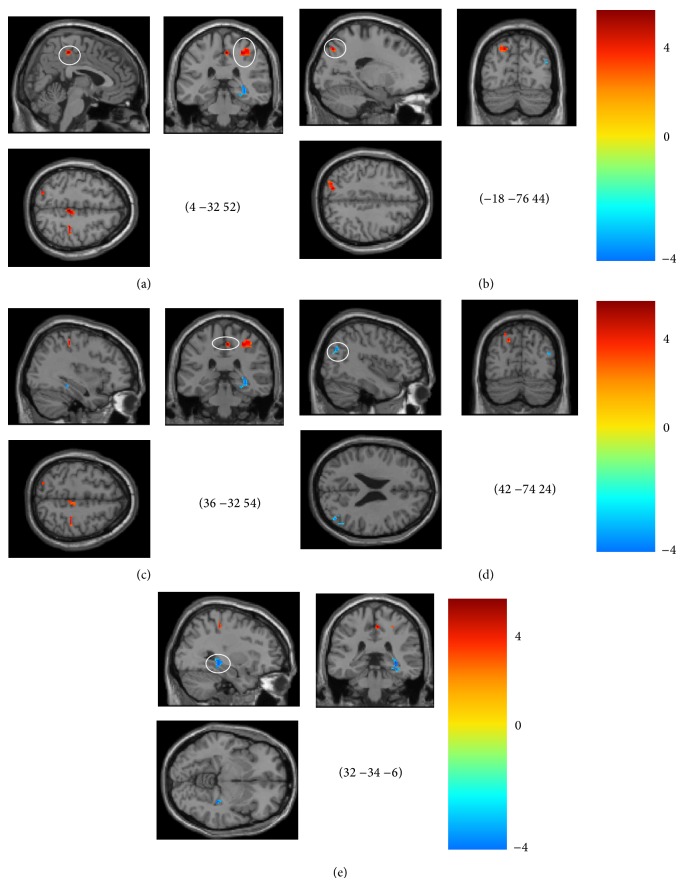
Brain areas with significant differences in the ReHo in the mild cognitive impairment (MCI) subjects (*P* < 0.01, cluster size larger than 50 voxels). (a), right paracentral Lobe; (b), left precuneus lobe; (c), right postcentral area; (d), right middle temporal gyrus; (e), right hippocampus.

**Figure 2:**
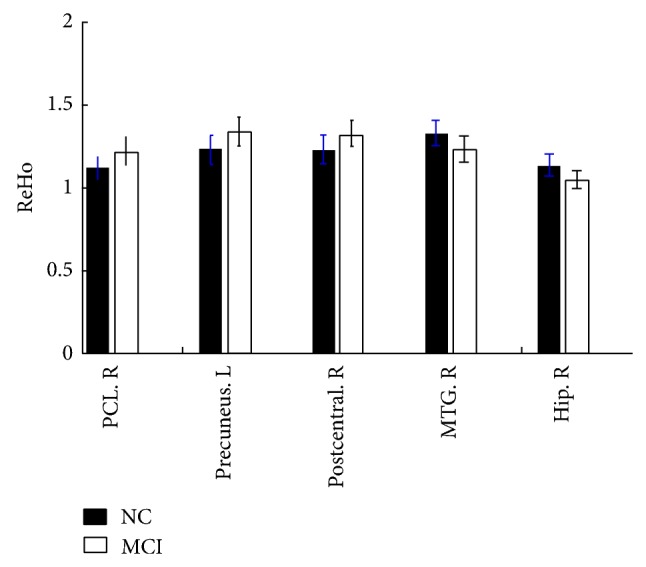
Bar of the mean ReHo index for the identified brain regions. All regions showed significant differences between the subjects with mild cognitive impairment (MCI) and the subjects with normal cognitive function. L: left; R: right; Hip: hippocampus; MTG: middle temporal gyrus.

**Figure 3:**
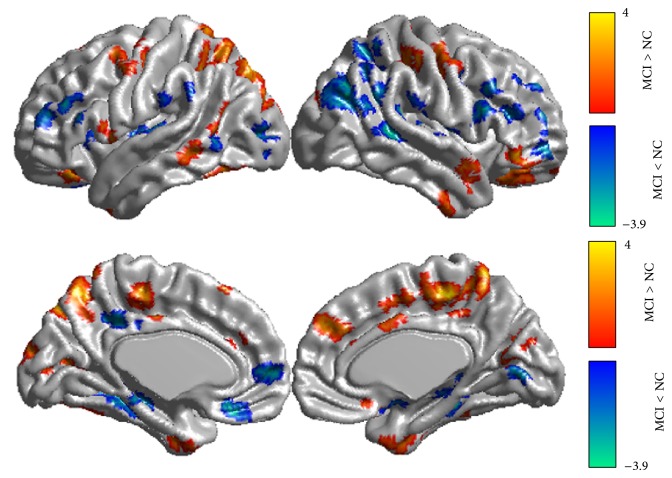
Brain areas with significant ReHo differences in the mild cognitive impairment (MCI) subjects (*P* < 0.05, cluster size larger than 50 voxels).

The spontaneous brain activity, which was measured with the ReHo index, was reduced in the patients with MCI. The ReHo index was significantly different in the following regions: the hippocampus, paracentral lobe, precuneus and medial temporal gyrus (MTG) (Figures 1 and 2). Many of the regions demonstrating significant between-group differences have previously been described as components of the default mode network when a loose statistical threshold was used in consideration of the slight impaired activity in MCI subjects (Figure 3).

For the identified brain regions, we found a positive trend in the correlation between the ReHo index in the precuneus and the MMSE scores (*P* = 0.058) in the patients with MCI; however, no significant correlations between the other ReHo indices and the clinical variables (e.g., MMSE and MoCA) were found in the MCI group.

## 4. Discussion

In the present study, significant differences in the ReHo indexs were found in various brain regions, including the paracentral, precuneus, postcentral, and medial temporal gyrus and the hippocampus in the MCI subjects compared with the subjects with normal cognitive function (Table 2, Figures 1 and 2). This finding aligns with previous related studies, even those that focused on searching for an altered ReHo measure in subjects with AD compared to subjects with normal cognitive function [17, 30, 31]. For example, Zhang and colleagues found spontaneous activations in the medial prefrontal cortex (MPFC), bilateral PCC/PCu and left inferior parietal lobule (IPL) of the brain were impaired in the MCI patients [30]; however, further analysis showed that the MCI subjects showed slight alterations in the ReHo index only in the left IPL compared to subjects with normal cognitive function. In another study, Bai and colleagues found increased ReHo indices in the right IPL, right fusiform gyrus, and bilateral putamen and decreased ReHo indices in the PCC/Pcu [29].

For the first time, decreased spontaneous activations in the hippocampus in MCI were observed, which aligns with reduced hippocampal activity inpatients with late aMCI and early AD [35, 36]. The hippocampal formation is the first brain structure to be affected by AD [37]. Numerous studies have consistently demonstrated hippocampal atrophy not only in patients with AD but also in subjects with MCI whose cognitive deficits are limited to the memory domain [38, 39]. Indeed, among dementia and amnesic patients, smaller hippocampi predict worse memory [40]. In the early stages of MCI, memory is worse than what would be expected for normal aging; these changes in hippocampal activation might reflect the structure atrophy and dysfunctional condition of this region in MCI subjects [41, 42]. Additionally, it should be noted that increased or decreased hippocampus activations in MCI patients have been obtained using resting-state fMRI [35, 36, 42, 43]. These discrepancies may be explained by a longitudinal study, which suggests that increased hippocampal activation commonly occurs in early stages of the disease and precedes decline in cognition and hippocampal activation [44]. The histopathological findings in the hippocampus in AD, the CA1 region in hippocampus has shown decreased metabolic responses [45]. Decreased activation within the hippocampal formation itself is believed to result in decreased activation of consecutive regions [46]. Then early AD pathology revealed by histopathological as well as neuroimaging studies might explain decreased activation within the hippocampus (mainly CA1) in MCI.

Again, most of the significantly different regions (e.g., the precuneus, the medial temporal gyrus, and hippocampus) are involved in the default mode network [6, 9]. The default mode network has been suggested as being engaged in internally focused tasks, including retrieving autobiographical memory and envisioning the future. In MCI, the default network has been found to be a sensitive hallmark of injury and is closely involved in episodic memory processing [47]. The precuneus, one of the key regions of the default network, is involved in detecting tactile and visual information and works in concert with the medial temporal lobe, which is tightly linked to long-term memory [48]. The MCI-related higher regional homogeneity may compensate for the damaged memory-related regions, such as the medial temporal gyrus (Figure 1), or for the decreased ReHo in the regions near the precuneus, such as the posterior cingulate cortex (Figure 3). Our study offered further evidence for MCI-related impairments in the default mode network, although the details of the mechanism of compensation require further research. During the memory encoding tasks, the precuneus might be deactivated rather than activated [49]. Neuropathological studies have found increased amyloid plaque deposition on the course of the AD continuum, morphological studies have found AD-like atrophy in the MCI and we found the different ReHo index. Taken together, the precunes might be prognostic to distinguish between high and low risk of conversion to AD.

Another interesting finding of the present study is that an increased ReHo index occurred in the postcentral and the paracentral lobes in the MCI subjects compared with the subjects with normal cognitive function (Table 2, Figures 1 and 2). The motor system changes may resemble those described for the cognitive network. It is common knowledge that the parietal cortex has extensive connections with the frontal lobe region, which could send rich sensory information for movement control [50]. Additionally, the frontal cortices are the key regions involved in human memory processing [9]. An increased ReHo index in the motor system might indicate overreaction of the sensorimotor network in MCI, which is consistent with the assumption that AD and MCI patients may be able to use additional neural resources in the prefrontal and surrounding regions to compensate for cognitive function losses [11, 51, 52]. However, we do not know if these alterations are compensatory to maintain good memory in MCI or if they are signs of impeding neuronal failure; therefore, more work is needed to study the function of the postcentral and the paracentral lobes in MCI patients.

The MMSE is one of the most widely used cognitive screening instruments for MCI, and it can be used to represent the global functioning of the subject. A positive correlation trend was found between the ReHo index in the precuneus and MMSE scores in the subjects with MCI (*P* = 0.058). Therefore, lower ReHo in the precuneus tends to be associated with lower MMSE scores. More subtle clinical typing (i.e., single-domain aMCI and multiple-domain aMCI) will be necessary to validate this finding.

One key limitation of the present results is that they do not exist after statistical correction. This outcome may occur because the MCI subjects have only very slight alterations in brain activity compared to the subjects with normal cognitive function; this observation is inconsistent with the results of a previous MCI study by Zhang and colleagues, which found significant alterations in AD in comparison with subjects with normal cognitive function [30]. Subjects with MCI showed less significant alterations. Another possible reason for this finding might be the individual variables in the MCI samples; not all subjects will progress to AD, although there is a high risk of progression. The exploratory analysis based on Gaussian mixture model suggested that the MCI subjects might be able to be subdivided into two groups (Figure S1 in supplementary materials available online at http://dx.doi.org/10.1155/2015/807093), which supports the role of the ReHo score as an early indicator. Hence, studies including large samples and longitudinal data are necessary.

## 5. Conclusion

The present study demonstrated that resting-state ReHo was impaired in the subjects with MCI. MCI subjects displayed higher ReHo indices in the paracentral and postcentral lobes and precuneus and lower ReHo indices in the hippocampus and medial temporal gyrus. These findings strengthened the theory that impaired brain activity in the default mode network could be an important marker for distinguishing MCI subjects.

## Supplementary Material

Figure S1: Results of Gaussian mixture distribution of the ReHo index in the selected two regions (left: PCL and precuneus; right: MTG and HiP).
Figure S2: Bar of the mean grey matter volume for the identified brain regions. No significant atropy were found in the identified brain regions (P>0.103).

## Figures and Tables

**Table 1:** Demographic and neuropsychological data in mild cognitive impairment (MCI) subjects and normal controls.

Characteristics	MCI	Controls	*P* value
	(*n* = 30)	(*n* = 32)	
Gender, female/male	12/18	17/15	0.30
Age, years	69.1 ± 5.8	70.1 ± 5.5	0.49
MMSE	26.2 ± 2.2	28.1 ± 1.5	<0.0001
MoCA	21.9 ± 3.4	26.3 ± 1.9	<0.0001
Head motion-move	0.42 ± 0.26	0.45 ± 0.38	0.70
Head motion-rotation	0.73 ± 0.56	0.79 ± 0.50	0.64

Chi-square was used for gender comparisons.

Two-tailed test was used for age, education, and neuropsychological test comparisons.

MMSE: Minimental state examination; MoCA: Montreal cognitive assessment.

**Table 2:** Altered ReHo scores in mild cognitive impairment (MCI) subjects in comparison to normal controls (*P* < 0.01, cluster size > 50 voxels).

Brain area	Cluster size	*T*-value	*Z*-value	MNI coordinates (*x*, *y*, *z*)		
Paracentral Lobe. R	53	4.95	4.5	4	−32	52

Precuneus. L	77	4.07	3.8	−18	−76	44
		3.24	3.09	−24	−72	52
		3.18	3.04	−28	−82	42

Postcentral. R	50	4.04	3.77	28	−32	52
		3.6	3.41	36	−32	54

MTG. R	73	−3.55	−3.36	50	−66	32
		−3.35	−3.19	40	−70	32
		−3.21	−3.07	44	−74	26

Hip. R	72	−3.67	−3.46	32	−34	−8
		−3.37	−3.20	30	−32	0

L: left; R: right; Hip: hippocampus; MTG: middle temporal gyrus.
