# Supplementary material for: Using Regional Homogeneity to Reveal Altered Spontaneous Activity in Patients with Mild Cognitive Impairment
Source: Biomed Res Int. 2015 Feb 9;2015:807093. doi: 10.1155/2015/807093 (PMC4337114; doi:10.1155/2015/807093)
Supplement: Supplementary file 1 — Figure S1: Results of Gaussian mixture distribution of the ReHo index in the selected two regions (left: PCL and precuneus; right: MTG and HiP). Figure S2: Bar of the mean grey matter volume for the identified brain regions. No significant atropy were found in the identified brain regions (P>0.103). [file 807093.f1.pdf]

Supplementary material

<u>ReHo score may be an early indicator for MCI</u>

Most patients with mild cognitive impairment (MCI) are thought to be in an early stage of Alzheimer's disease (AD). Approximately 50% of patients with MCI, particularly amnestic MCI, will develop Alzheimer's disease (AD) within 3–5 years [1, 2]. Then 50% of MCI patients will deteriorate, these two groups (converters and non-converters) should be contained in their patients's cohort. So we explored the homogeneity of the distribution of the ReHo score in the MCI groups by using Gaussian mixture models for the ReHo indices of the identified regions. For the sample size is relative small (only 32 MCI subjects totally), so we random selected combine two regions indices to be a two-dimension scores. The Gaussian mixture distribution results suggested that there might exist two sub-classes in the MCI subjects (Figure S1).

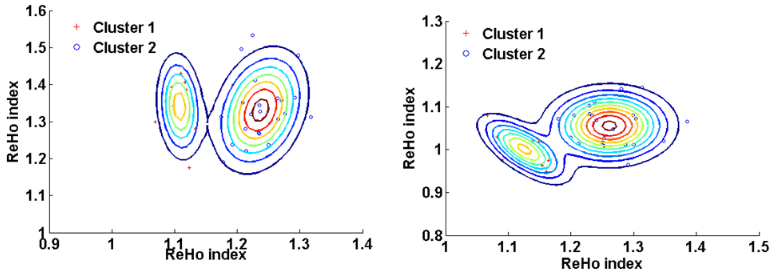

Figure S1. Results of Gaussian mixture distribution of the ReHo index in the selected two regions (left: PCL and precuneus; right: MTG and HiP).

The exploratory analysis based on Gaussian mixture model suggested that the MCI subjects might be able to be subdivided into two groups (Fig S1), which support the role of the ReHo score as an early indicator.

<u>No significant atropy were found in the identified brain regions in the MCI sujects</u>

The increase of the ReHo score in some midline areas which is somehow unexpected and in contrary to the majority of previous reports. We explored whether the relation between the cognitive scores and the ReHo score of the midline areas reaches statistical significance after correction for hippocampal atrophy. Then, we performed the analysis and our results did not showed strong correlation between the MMSE and the ReHo index in the identified brain regions. Also we have evaluated the grey matter volumes of the identified regions. Our results demonstrated that there is no significant atrophy ($P>0.103$) of the identified brain regions even these regions showed altered functional connectivities.

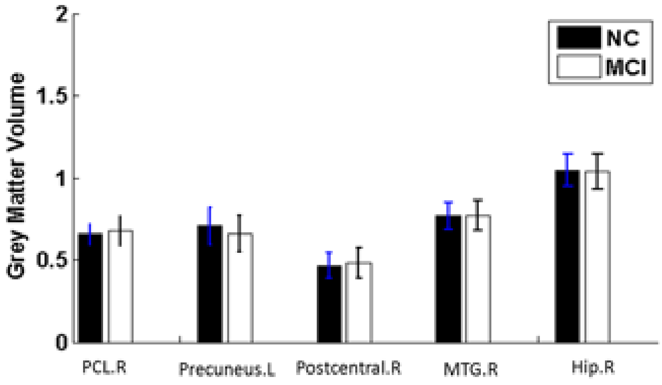

Fig. S2 Bar of the mean grey matter volume for the identified brain regions. No significant atropy were found in the identified brain regions (P>0.103).

References:

1. Petersen RC, Smith GE, Waring SC, Ivnik RJ, Tangalos EG, et al. (1999) Mild cognitive impairment: clinical characterization and outcome. Arch Neurol 56: 303-308.

2. Petersen RC, Doody R, Kurz A, Mohs RC, Morris JC, et al. (2001) Current concepts in mild cognitive impairment. Arch Neurol 58: 1985-1992.
